# Potential Pathogenicity and Genetic Characteristics of a Live-Attenuated Classical Swine Fever Virus Vaccine Derivative Variant

**DOI:** 10.1155/2024/7244445

**Published:** 2024-04-12

**Authors:** Zhenhua Guo, Guangxu Xing, Leyi Wang, Qianyue Jin, Qingxia Lu, Gaiping Zhang

**Affiliations:** ^1^Key Laboratory of Animal Immunology of the Ministry of Agriculture, Henan Provincial Key Laboratory of Animal Immunology, Institute for Animal Health, Henan Academy of Agricultural Sciences, Zhengzhou, China; ^2^Department of Veterinary Clinical Medicine and the Veterinary Diagnostic Laboratory, College of Veterinary Medicine, University of Illinois at Urbana-Champaign, Urbana, IL, USA; ^3^Longhu Modern Immunity Laboratory, Zhengzhou, Henan, China; ^4^School of Advanced Agricultural Sciences, Peking University, Beijing, China

## Abstract

Classical swine fever (CSF), caused by CSF virus (CSFV), is a highly contagious disease affecting pigs and causing massive pig production losses with severe global economic recession. The immunization of live-attenuated vaccines is still one of the key measures to CSFV management in endemic countries. However, there are also strong controversies about the usage of live-attenuated vaccines, particularly in pregnant sows and young pigs, such as in Europe, where domestic pigs are routinely not vaccinated until severe outbreaks occur. Here, we report a CSF outbreak in a pig farm in China, which affected more than 90% of the delivery sows and led to ∼45% birth loss. Surprisingly, phylogenetic analysis showed that the CSFV isolate (named CSFV/HeNLY2022, GenBank No. OR195698) was clustered into subgenotype 1.1a, closely together with the live-attenuated vaccine strains. Further genomic analysis also revealed that the isolate CSFV/HeNLY2022 shared the highest nucleotide identity of 99.7% with the C/HVRI vaccine strain (C-strain, GenBank No. AY805221). Moreover, compared to the C/HVRI strain, a total of eight amino acid mutations, distributed in E^rns^ (H436^th^Y and S476^th^R), E1 (T502^th^I and P581^th^T), E2 (M979^th^K and A1061^th^S), NS5A (A2980^th^T), and NS5B (I3818^th^M), were characterized in the CSFV/HeNLY2022 isolate. Our results suggested that the CSF outbreak was most likely caused by the live-attenuated CSFV vaccine or its derivative. It raises concern that the unscientific application of CSFV vaccines could potentially lead to CSFV spread in pigs. It is needed to perform a more rigorous evaluation of the safety of the C-strain-derived vaccines in combination with other different live-attenuated vaccines.

## 1. Introduction

Classical swine fever (CSF) remains one of the most economically important viral diseases to pigs worldwide and is notifiable to the World Organization for Animal Health [1]. The causative agent, CSF virus (CSFV), belongs to the genus *Pestivirus* (*Pestivirus suis* species) in the *Flaviviridae* family and is an enveloped, single-stranded, positive-sense RNA virus [2, 3]. Similar with the other members of the *Pestivirus* genus, the genome size is about 12.3 kb with 5′ untranslated region (5′ UTR), one open reading frame encoding a single polyprotein, and 3′ UTR. The polyprotein is further cleaved by host or viral proteases into four structural proteins (Core, E^rns^, E1, E2) and eight nonstructural proteins (Npro, P7, NS2, NS3, NS4A/B, NS5A/B) [4]. Based on the sequence of 5′ UTR, E2, and NS5B, CSFVs are genetically divided into three genotypes (1, 2, and 3) and at least 11 subgenotypes (1.1–1.4, 2.1−2.3, and 3.1–3.4) [1, 2]. Genotype 2 is predominantly circulating in Asia and Europe [4–6].

Currently, there are approximately 38 CSFV-free countries, including all of North America, Oceania, as well as a large part of the European Union [2]. However, CSF is still a problematic issue for most countries of Asia, South and Central America, and the Caribbean [4]. Several live-attenuated vaccines, such as the lapinized Chinese vaccine (also known as C-strain), the Japanese guinea-pig exaltation-negative (GPE−) strain, and the French cell culture adapted Thiverval strain, have been widely applied in mandatory control programs in CSFV endemic countries [7, 8]. Although these vaccines are generally safe and effective, a strong controversy also exists, particularly in pregnant sows and young pigs, such as in Europe, where domestic pigs are not routinely vaccinated until severe outbreaks occur [4].

## 2. Materials and Methods

From July 20, 2022, a severe reproductive disorder broke out in a pig farm in Henan province, China. To explore the causative agents, clinical specimens, including seven tissues from aborted and mummified fetuses, three serums from aborted sows, and two semen from boars, were delivered to the laboratory for pathogen detection. The test methods for CSFV, porcine reproductive and respiratory syndrome virus (PRRSV), porcine pseudorabies virus (PRV), porcine parvovirus (PPV), Japanese encephalitis virus (JEV), porcine circovirus 2 (PCV2), and porcine circovirus 3 (PCV3) were according to a previously described methods [9–11].

Viral nucleic acid was extracted from samples using MiniBEST Viral RNA/DNA Extraction Kit Ver.5.0 (TaKaRa, Code No.9766). Viral RNA was further reverse transcribed to cDNA using PrimeScript™ RT reagent Kit (TaKaRa, Code No. RR037Q). The harvested viral DNA and cDNA were stored at −80°C for further studies. To determine the genetic evolution of the CSFV strain in the pig farm, 13 pairs of primers were designed (reference strain Genbank no. AF091507) to amplify the genomic sequence (Table S1).

The evolutionary relationship was analyzed by MEGA 11.0 software using the neighbor-joining method with the Kimura 2-parameter model [12]. The genomic homology analysis and amino acid sequence alignment were performed using the MegAlign program of DNASTAR package (DNASTAR, Inc., Madison, WI, USA) to determine sequence homology and genetic variations. And genomic similarity was further evaluated using SimPlot 3.5.1 software [13]. The reference CSFV strain information is listed in Table S2.

## 3. Results and Discussion

**Figure 1:**
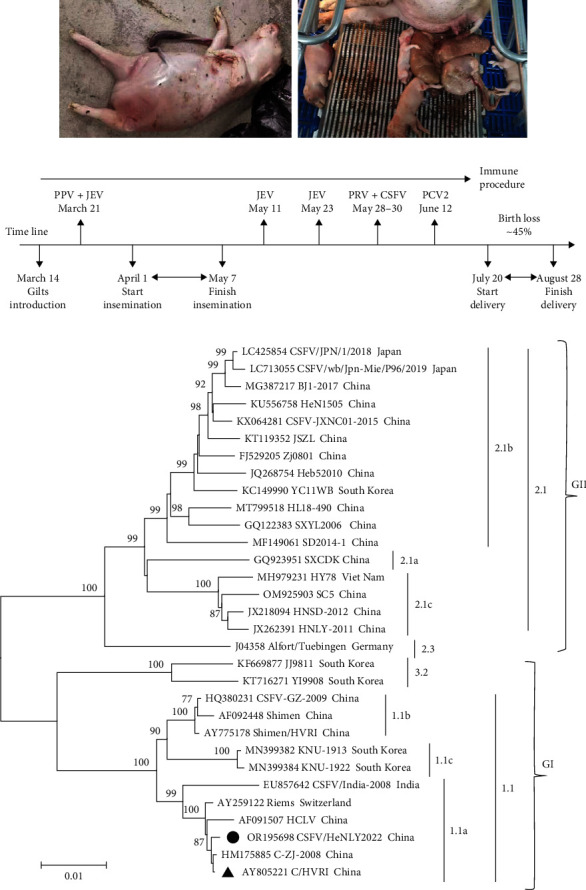
The clinical information of the pig farm and phylogenetic analysis of the CSFV isolates. (a) Mummies, rotten, and white fetuses were observed when the sows began to deliver. (b) The immune procedure in the pig farm. PPV: WH-1 strain, inactivated vaccine; JEV: SA14-14-2 strain, live-attenuated vaccine; PRV: Batha-K61 strain, live-attenuated vaccine; CSFV: cell culture adapted C-strain, live-attenuated vaccine; PCV2: recombinant vaccine. (c) Phylogenetic analysis based on polyprotein nucleotide sequences of CSFVs. The phylogenetic tree was inferred using MEGA 11.0 with the neighbor-joining method. The CSFV isolate CSFV/HeNLY2022 was marked by a black solid circle (●). The live-attenuated prototype strain C/HVRI (GenBank no. AY805221) was indicated by a triangle symbol (▲).

During July 20–August 28, 2022, severe porcine reproductive disorders occurred at a pig farm in Henan province, China (Figure 1(a)). Clinical specimens, including seven tissues (spleen, inguinal lymph nodes, tonsil, and kidney) from stillbirths, newborn piglets, three serums from aborted sows, and two semen samples obtained from boars, were sent to a laboratory for pathogen detection on July 27, 2022. All samples were tested negative for PRRSV, PRV, PPV, and JEV by virus-specific nucleic acids tests. Serum from only one sow tested positive for PCV2 and PCV3. However, 10 of the 12 specimens were CSFV positive, and one semen sample was suspected for CSFV. Then, a survey was conducted by consulting with veterinarians on the pig farm. On March 14, the farm introduced 123 gilts (about 125 kg) from a professional breeding farm, where the pigs are free of several diseases, including CSFV, PRRSV, PRV, JEV, and PPV. The insemination was completed from April 1 to May 7. Meanwhile, live-attenuated vaccines were applied following the immune procedure, including JEV (SA14-14-2 strain) immunized on May 11 and 23, CSFV (cell culture adapted C-strain derivatives) and PRV (Batha-K61 strain) simultaneously immunized during May 28–30 (Figure 1(b)). Interestingly, the sows did not present any abnormal clinical symptoms until delivery. When the sows began to deliver, a large number of mummies, rotten, and white fetuses were observed, accounting for ∼33.0% of the newborn piglets, and the mortality rate of living piglets reached about 10% within 7 days. Overall, approximately 90% of the delivery sows showed different degrees of reproductive disorders, and the birth loss was about 45%.

**Figure 2:**
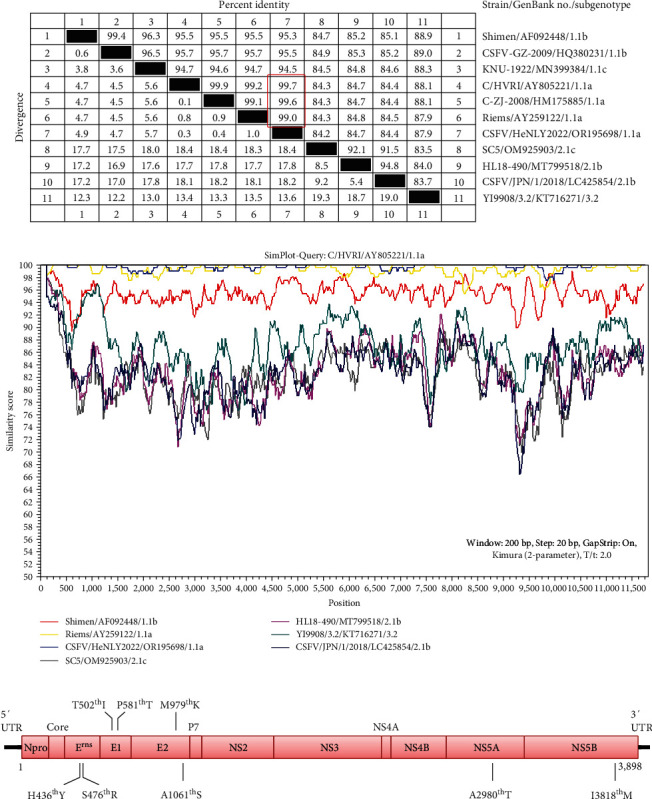
Homological analysis of the CSFV isolates. (a) The genomic homology analysis between the isolate CSFV/HeNLY2022 and reference strains. The red rectangular box showed the percent indentity of CSFV/HeNLY2022 with live-attenuated strains. (b) Sequence similarity was compared between CSFV/HeNLY2022 and representative CSFV strains. Sequence similarity was performed using the live-attenuated vaccine C/HVRI strain as the query. (c) Amino acid mutation analysis between C/HVRI strain and CSFV/HeNLY2022 isolate.

High genetic variability is a common feature of the CSFV genome [1]. To determine the genetic evolution of the CSFV strain in the pig farm, 13 pairs of primers were used to amplify the genomic sequence (Table S1). A near complete genome of the strain (named CSFV/HeNLY2022, GenBank No. OR195698) was acquired except the sequence of 478 bp at the 3′UTR. Based on the encoding sequence of the polyprotein, a phylogenetic tree was constructed by the neighbor-joining method using MEGA11.0. As shown in Figure 1(c), different with traditional CSFV classification, the CSFVs could be divided into two evolutionary groups-GI (includes genotypes 1 and 3) and GII (mainly genotype 2), consistent with the classification of a previous study [4]. Surprisingly, CSFV/HeNLY2022 was clustered into subgenotype 1.1a, closely together with the live-attenuated vaccine strains. Further genomic similarity analysis also revealed that the CSFV/HeNLY2022 strain shared the highest nucleotide identity of 99.7% with the C/HVRI vaccine strain (C-strain/Harbin Veterinary Research Institute, GenBank No. AY805221), but lower identities 94.5%−95.3% with the reference genotype 1.1b and 1.1c strains, and 84.2%−87.9% with the reference genotype 2.1 and 3.2 strains (Figures 2(a) and 2(b)). Moreover, compared to the C/HVRI strain, a total of eight amino acid mutations were found in the CSFV/HeNLY2022 isolate. As shown in Figure 2(c), the substitutions are distributed in E^rns^ (H436^th^Y and S476^th^R), E1 (T502^th^I and P581^th^T), E2 (M979^th^K and A1061^th^S), NS5A (A2980^th^T), and NS5B (I3818^th^M). Taken together, these data suggest that CSFV/HeNLY2022 is a vaccine-derivative strain.

C-strain, also known as C/HVRI, HCLV (hog cholera virus lapinized Chinese vaccine strain, HCLV) (GenBank No. AF091507 and AF531433), was jointly developed by China Institute of Veterinary Drugs Control and Harbin Veterinary Research Institute (HVRI) in China in 1950s and is by far the most frequently used CSF vaccine strain [8]. Based on the C-strain, several modified live vaccines, such as C-strain Riems, C-strain cell line origin, and Ingelvac® CSF MLV, were further developed to improve the safety and efficacy of protection. These live vaccines played a critical role in the control and eradiation of global CSF. Generally, C-strain is genetically stable and safe for pigs of all ages [14], and extensive use data exist mainly for the C-strain vaccines. However, the application data (for example, the genetic variation after cell-adapted culture) of modified live vaccines, particularly the cell line origin, is relatively few.

Here, we observed a severe porcine reproductive disorder in a pig farm in China. Laboratory diagnosis (10 of the 12 clinical samples were CSFV positive) and genetic analysis showed that the pathogenic agent might be CSFV, and the strain sequence has high genomic homology (99.7%) with swine fever attenuated vaccine strain (C/HVRI, GenBank No. AY805221). To the best of our knowledge, this is the first report indicating that CSFV live vaccines (cell culture-adapted C-strain derivatives) may have the potential to induce pathogenicity in immunized pigs under specific conditions. Meanwhile, it is not possible to exclude the conclusion that other unidentified agents or factors could have resulted in the reproductive issues observed (the possibility of this scenario is considered low, since most of the clinical samples were CSFV positive), until the virus has been isolated and clinical disease reproduced by experimental infection.

One possible reason for the reproductive failures could be the intensive nature and timings of the immunization. The pregnant sows had been administered two doses of the JEV live-attenuated vaccine (17 and 5 days ago) before immunized simultaneously with PRV and CSFV live-attenuated vaccines. The attenuated JEV and PRV vaccine strain could change the immune status of pregnant sows, which might make them more susceptible to CSFV. On the other hand, the amino acid substitutions in the polyprotein region also perhaps led to the virulence change. In the future, it is necessary to further explore whether these mutations are associated with the altered pathogenicity of the C-strain vaccine by virus isolation, reverse genetics systems and pig challenge studies. Another disadvantage of the traditional CSFV live vaccines is incompatible with a serological differentiation of infected from vaccinated animals (DIVA), which limits the CSF eradication in many countries [5]. Thus, many efforts have been put into develop novel effective DIVA vaccines. Recently, E2 subunit vaccines have been developed and authorized in Europe and China, which would be very useful to promote the prevention and eradication of CSFV [5, 15, 16].

In conclusion, our study suggests that the live-attenuated CSFV vaccine will most likely cause clinical diseases in sows and raises concern that the unscientific application of CSFV live vaccines in pig immunization procedures could potentially spread CSFV in pig farms. It is needed to perform a more rigorous evaluation of the safety of the C-strain-derived vaccines in combination with different live-attenuated vaccines. Further monitoring of combined vaccination application in pigs is also urgently needed.

## Data Availability

All data generated or analyzed during this study are included in this published article and its supplementary information files.

## Supplementary Materials

Supplementary Materials Table S1: primers used for genome sequencing. Table S2: the reference sequence information in this study.

## References

[1] Zhou B. (2019). Classical swine fever in China—an update minireview. *Frontiers in Veterinary Science*.

[2] Ganges L., Crooke H. R., Bohórquez J. A. (2020). Classical swine fever virus: the past, present and future. *Virus Research*.

[3] Postler T. S., Beer M., Blitvich B. J. (2023). Renaming of the genus flavivirus to orthoflavivirus and extension of binomial species names within the family Flaviviridae. *Archives of Virology*.

[4] Liu Y., Bahoussi A. N., Wang P.-H., Wu C., Xing L. (2022). Complete genome sequences of classical swine fever virus: phylogenetic and evolutionary analyses. *Frontiers in Microbiology*.

[5] Postel A., Austermann-Busch S., Petrov A., Moennig V., Becher P. (2018). Epidemiology, diagnosis and control of classical swine fever: recent developments and future challenges. *Transboundary and Emerging Diseases*.

[6] Postel A., Nishi T., Kameyama K.-I. (2019). Reemergence of classical swine fever, Japan, 2018. *Emerging Infectious Diseases*.

[7] Leifer I., Depner K., Blome S. (2009). Differentiation of C-strain “Riems” or CP7_E2alf vaccinated animals from animals infected by classical swine fever virus field strains using real-time RT-PCR. *Journal of Virological Methods*.

[8] Luo Y., Li S., Sun Y., Qiu H.-J. (2014). Classical swine fever in China: a minireview. *Veterinary Microbiology*.

[9] Guo Z., Li X., Deng R., Zhang G. (2019). Detection and genetic characteristics of porcine circovirus 3 based on oral fluids from asymptomatic pigs in central China. *BMC Veterinary Research*.

[10] Wu H., Rao P., Jiang Y., Opriessnig T., Yang Z. (2014). A sensitive multiplex real-time PCR panel for rapid diagnosis of viruses associated with porcine respiratory and reproductive disorders. *Molecular and Cellular Probes*.

[11] Xu X.-G., Chen G.-D., Huang Y. (2012). Development of multiplex PCR for simultaneous detection of six swine DNA and RNA viruses. *Journal of Virological Methods*.

[12] Tamura K., Stecher G., Kumar S., Battistuzzi F. U. (2021). MEGA11: molecular evolutionary genetics analysis version 11. *Molecular Biology and Evolution*.

[13] Lole K. S., Bollinger R. C., Paranjape R. S. (1999). Full-length human immunodeficiency virus type 1 genomes from subtype C-infected seroconverters in India, with evidence of intersubtype recombination. *Journal of Virology*.

[14] Hao G., Zhang H., Chen H., Qian P., Li X. (2020). Comparison of the pathogenicity of classical swine fever virus subgenotype 2.1c and 2.1d strains from China. *Pathogens*.

[15] Chen J.-Y., Wu C.-M., Chen Z.-W. (2021). Evaluation of classical swine fever E2 (CSF-E2) subunit vaccine efficacy in the prevention of virus transmission and impact of maternal derived antibody interference in field farm applications. *Porcine Health Management*.

[16] Chen W.-T., Liu H.-M., Chang C.-Y. (2023). Cross-reactivities and cross-neutralization of different envelope glycoproteins E2 antibodies against different genotypes of classical swine fever virus. *Frontiers in Veterinary Science*.

