# Supplementary material for: Potential Pathogenicity and Genetic Characteristics of a Live-Attenuated Classical Swine Fever Virus Vaccine Derivative Variant
Source: Transbound Emerg Dis. 2024 Apr 12;2024:7244445. doi: 10.1155/2024/7244445 (PMC12016863; doi:10.1155/2024/7244445)
Supplement: Supplementary Materials — Table S1: primers used for genome sequencing. Table S2: the reference sequence information in this study. [file 7244445.f1.docx]

**Table S1.** Primers used for genome sequence

| **Primer name** | **Sequence 5’-3’** |
| --- | --- |
| P1-For | GTATACGAGGTTAGTTCATTCT |
| P1-Rev | AATAGGGCTTTTTCTAGTTTCTTCC |
| P2-For | AAGAAGCCAGATAGGATCAACAAAG |
| P2-Rev | GAAAGCAGCAGAAATTCTGATAGGT |
| P3-For | CTTATGCCCTATCGCCTTACTGT |
| P3-Rev | GGCTCGCCTTTCACACATGT |
| P4-For | GGGTGGACGGGTGTCATAGAGT |
| P4-Rev | TAAGGTACTGTAGCCAGGTCTTGTATC |
| P5-For | GCTGGCAAAGAGAGATCCGACT |
| P5-Rev | TGGGAGATTCCTCAGGAACAATT |
| P6-For | ACCTGTGGCATGACCCTAGC |
| P6-Rev | TCAAGAAATATGAAGGAGTACTCAACC |
| P7-For | GCGGCAGCAGAGTCAGTATACC |
| P7-Rev | CCCCTAATTTTCTTGCGTTGAG |
| P8-For | AATAATTTGTTGATATCAGATGAACTACC |
| P8-Rev | GCCCCTACCCCTAGCATGAC |
| P9-For | CCGATTGGAGAGCGTTGTCAT |
| P9-Rev | GTGTAAGCCAGCCAGGTTGTAAC |
| P10-For | TGATAGAGAGGGACTGTGCAACC |
| P10-Rev | CGGGACACAGTGTTTTCATCTTG |
| P11-For | AACAAAACTGCACACATGGTCTCT |
| P11-Rev | GCCATCATCCCCGCACAC |
| P12-For | TGCTGATGGGGAAGTATACATAAGG |
| P12-Rev | GCAAAGAGGCAGTGGACAACC |
| N12-For | CACCTATATTGACACAGTCTTGTAGTAGT |
| N12-Rev | GCCCTCTTTAACACCTATATTGACAC |

**Table S2.** The reference sequence information in this study**.**

| No. | Strain name | Country | Genbank no. | Year | Subgenotype |
| --- | --- | --- | --- | --- | --- |
| 1 | SXYL2006 | China | GQ122383 | 2006 | 2.1b |
| 2 | SXCDK | China | GQ923951 | 2009 | 2.1a |
| 3 | HNSD-2012 | China | JX218094 | 2012 | 2.1c |
| 4 | YC11WB | South Korea | KC149990 | 2011 | 2.1b |
| 5 | HeN1505 | China | KU556758 | 2015 | 2.1d |
| 6 | CSFV-JXNC01-2015 | China | KX064281 | 2015 | 2.1b |
| 7 | HY78 | Viet Nam | MH979231 | 2015 | 2.1c |
| 8 | KNU-1913 | South Korea | MN399382 | 2019 | 1.1c |
| 9 | KNU-1922 | South Korea | MN399384 | 2019 | 1.1c |
| 10 | Zj0801 | China | FJ529205 | 2008 | 2.1b |
| 11 | HNLY-2011 | China | JX262391 | 2011 | 2.1c |
| 12 | JSZL | China | KT119352 | 2014 | 2.1b |
| 13 | HL18-490 | China | MT799518 | 2018 | 2.1b |
| 14 | Alfort/Tuebingen | Germany | J04358 | 1989 | 2.3 |
| 15 | CSFV/JPN/1/2018 | Japan | LC425854 | 2018 | 2.1b |
| 16 | BJ1-2017 | China | MG387217 | 2017 | 2.1b |
| 17 | Heb52010 | China | JQ268754 | 2010 | 2.1b |
| 18 | SD2014-1 | China | MF149061 | 2014 | 2.1b |
| 19 | C/HVRI | China | AY805221 | 1954 | 1.1a |
| 20 | CSFV-GZ-2009 | China | HQ380231 | 2009 | 1.1b |
| 21 | Shimen/HVRI | China | AY775178 | 2004 | 1.1b |
| 22 | Shimen | China | AF092448 | 1945 | 1.1b |
| 23 | C-ZJ-2008 | China | HM175885 | 2008 | 1.1a |
| 24 | CSFV/India-2008 | India | EU857642 | 2008 | 1.1a |
| 25 | Riems | Switzerland | AY259122 | 2003 | 1.1a |
| 26 | JJ9811 | South Korea | KF669877 | 1998 | 3.2 |
| 27 | YI9908 | South Korea | KT716271 | 1999 | 3.2 |
| 28 | SC5 | China | OM925903 | 2020 | 2.1c |
| 29 | CSFV/wb/Jpn-Mie/P96/2019 | Japan | LC713055 | 2019 | 2.1b |
| 30 | HCLV | China | AF091507 | 1998 | 1.1a |
| 31 | CSFV/HeNLY2022 | China | OR195698 | 2022 | 1.1a |
